# Rats Born to Mothers Treated with Dexamethasone 15 cH Present Changes in Modulation of Inflammatory Process

**DOI:** 10.1155/2012/710923

**Published:** 2012-07-29

**Authors:** Leoni V. Bonamin, Cristiane Landi de Moraes, Fernanda Sanches, Thayná Neves Cardoso, Cesar Sato, Claudemir Duran Filho, Lucienne C. Martini

**Affiliations:** ^1^Laboratory of Cell and Molecular Biology, Research Center, Universidade Paulista (UNIP), São Paulo, SP, Brazil; ^2^Laboratory of Veterinary Pathology, Universidade de Santo Amaro (UNISA), São Paulo, SP, Brazil; ^3^Royal Technology and Development Institute, São Roque, SP, Brazil

## Abstract

As little information about the effect of ultra high dilutions of glucocorticoid in reproduction is available in the literature, pregnant female Wistar rats (*N* = 12) were blindly subcutaneously treated during all gestational and lactation period with: dexamethasone 4 mg/kg diluted into dexamethasone 15 cH (mixed); or dexamethasone 4 mg/kg diluted in water; or dexamethasone 15 cH, or vehicle. Parental generation had body weight, food and water consumption monitored. The F1 generation was monitored regarding to newborn development. No birth occurred in both groups treated with dexamethasone 4 mg/kg. After 60 days from birth, 12 male F1 rats were randomly selected from each remaining group and inoculated subcutaneously with 1% carrageenan into the footpad, for evaluation of inflammatory performance. Edema and histopathology of the footpad were evaluated, using specific staining methods, immunohistochemistry and digital histomorphometry. Mothers treated with mixed dexamethasone presented reduced water consumption. F1 rats born to dexamethasone 15 cH treated females presented significant increase in mast cell degranulation, decrease in monocyte percentage, increase in CD18+ PMN cells, and early expression of ED2 protein, in relation to control. The results show that the exposure of parental generation to highly diluted dexamethasone interferes in inflammation modulation in the F1 generation.

## 1. Introduction

In the attempt to characterize the high-dilution action as a biological phenomenon, several experimental studies have been performed in the last years using cell or animal models [1–15] or even vegetal models [16–20], giving experimental and theoretical substrate to the development of agricultural tools using high dilutions of active substances, that is called “agro-homeopathy”. These studies include clinical veterinary research in livestock animals, and the homeopathic medicines point out as possibilities to treat common diseases or serve as zoo technical tools [21–25]. In Brazil, several trade homeopathic products are in use for these purposes nowadays; they are offered to animals as water and food additives to improve milk, egg, and meat production. Generally, no restriction is made to pregnant females, since no chemical residues are expected in animal-derived products.

In face to this contemporaneous scenario, the aim of this study was to create an experimental model to check if the exposure of mothers to high dilutions of a biological active substance is able to produce changes in reproduction and hereditary features in the offspring.

In previous studies made by our group, the isopathy principle was checked in laboratory animals using dexamethasone as an experimental model. Dexamethasone was chosen because of its well-known pharmacological action, whose results could be easily identified. Also, as a corticoid hormone, it could be used in a wide variety of experimental models and results could be compared to each other [1, 26].

In this study, the dexamethasone model was used because of the availability of its background data and because of its known action on reproduction [27–29]. Moreover, we should use a referenced experimental model to evaluate the safe of pregnant rat to homeopathic/isopathic substances and some putative physiological interference in offspring. The elucidation of this knowledge is fundamental, regarding all possibilities in basic research about high dilutions and also the establishment of further guidelines to the regulation of products used in organic agriculture.

## 2. Methods

### 2.1. Animals

Adult 60 days old female Wistar rats were supplied by the UNITOX-Royal Technology and Development Institute. One week before the beginning of the experiment, they were randomly organized in conventional polypropylene cages, receiving water and food *ad libitum*. Animals were maintained in controlled SPF environmental conditions during all experimental period, being temperature about 22 ± 2°C and humidity about 70%, with a regular light-dark cycle of 12 : 12 hours. Regular chemical and microbiological analysis of food and water was systematically made. The manipulation of all animals was made exclusively by a specific and authorized technician during all experimental period. All experimental data and environmental registers were recorded and checked by an independent observer, according to the Brazilian GLP (good laboratory practices) rules. No environmental extraordinary event was noticed.

### 2.2. Groups

P (parents) generation females were randomly distributed in four groups of 12 animals each. During *in vivo* manipulation, each group received a specific nonidentifiable code (CR), in order to warrant blindness:

 CR1: treated with dexamethasone 4 mg/kg diluted into dexamethasone 15 cH (MIX),
 CR2: treated with dexamethasone 4 mg/kg (DEX, positive control),
 CR3: treated with dexamethasone 15 cH (UHD, ultra-high dilution),
 CR4: vehicle or water (CONT, negative control).

### 2.3. Blind

Group codes were made by a person who was not directly related to the experimental manipulation and were maintained closed in a sealed cover up to the final statistics, after all procedures of cell counting.

Treatments were done three times a week, by subcutaneous injection [1], in a volume equal to 0.1 mL/100 g of weight, during all gestation (21 days) and lactation (20 days) period. Males were used only for mating.

### 2.4. Drugs

 (a) *Dexamethasone 15 cH*. The dilutions were prepared in a pharmacy certified by ANVISA (Brazilian National Agency of Sanitary Vigilance), according to the Brazilian Homeopathic Pharmacopeia. The matrix used was the commercial formula Decadron, which was serially diluted 1 : 100 in 10 mL of sterile water in the first 14 steps of homeopathic dilutions (14 CH). The last dilution/succussion step was made at the moment of administration, also using 10 mL of sterile water, respecting the same method used in previous steps. The final solution reached the theoretical concentration of 10^−33^ M. All dilutions were made in standard glass vials, as defined by the Brazilian Homeopathic Pharmacopeia.
 (b) *Dexamethasone 4 mg/kg*. The commercial formula Decadron was used in the dose of 4 mg/kg, diluted in sterile water and not submitted to succussions.
 (c)* Mix Dexamethasone 15  cH + 4 mg/kg*. In this case, the commercial Decadron was used in the same manner as described above, but, instead of using sterile water to prepare the solution, the dexamethasone 15 cH prepared some minutes before, according to (a), was used as solvent.

The dilution 15 cH was chosen because, in clinical practice, it can be used to treat a wide range of symptoms, from those of behavioral nature up to those related to functional and structural features. In fact, in this protocol, the behavior development and the inflammation process of subcutaneous tissue were both analyzed and related in the F1 (first descendent) generation. Also, data about the effects of dexamethasone 15 cH in acute experimental inflammation were well described before [1]. Beside these facts, the 15 cH dilution reaches the final theoretical concentration of 10^−33^ M which is above Avogadro's number, thus, represents a good challenge to check the effects of ultra high dilutions on mothers and offspring.

The pure water (vehicle) was chosen as negative control because, as observed in a systematic review [2], contrary to what is observed *in vitro*, the succussion of water seems to be irrelevant in studies about high dilutions performed *in vivo*.

### 2.5. Experimental Design

*Day Zero of Gestation*. It was determined from vaginal smears and the observation of spermatozoids. Couples of males and females were kept together during 3 weeks. Females that did not get pregnant during this period were withdrawn. During gestation, cage food and water consumption, as well as individual weigh gain, were registered.

*Day Twenty of Gestation*. It was the expected day for birth. Late or precocious births were registered, as well as the number of male and female newborns and the individual weight of each young rat. From the first day of life, only four males and four females by offspring were randomly chosen to be kept with their mothers, in order to avoid putative food competition in case of crowded cages. The observation of individual postpartum development was made using these selected animals.

### 2.6. Postpartum Development

From the birth, the F1 generation was analyzed according the following variables:

1. daily weight individual control,
2. day of fur appearance,
3. day of eyes opening,
4. day of pinna detachment,
5. day of first incisive tooth outbreak,
6. day of testicles dehiscence in males,
7. day of vagina opening in females,
8. day of complete development of postural reflex.

The postural reflex was evaluated by the capacity of newborn to return immediately to the quadruped position after been placed in dorsal *decubitus.* The definitive learning of this reflex is determined in the third consecutive day in which the newborn is able to restore immediately the stand position.

After the weaning, the P generation females were euthanized by anesthesia overdose and submitted to necropsy.

### 2.7. Inflammation Analysis

After 60 days of life, 12 newborns (F1 generation), from different mothers of the same group, were randomized in two groups, UHD and CONT, and submitted to subcutaneous injection of 0.1 mL of 1% *kappa* carrageenan (SIGMA) to induction of inflammatory edema, which was measured during 4 hours by micrometry (Mytutoyo). The estimated volume of the paw was calculated according to [1]. After 4 hours of observation, animals were sacrificed and the foot pad was processed according to the conventional histological procedures and stained by hematoxylin-eosin method (HE) for setting morphological references and counting polymorphonuclear cells (PMN) or neutrophils. A complementary staining method—toluidin blue—was used in order to analyze and quantify the percentage of degranulated mast cells/basophiles per field, by digital histomorphometry, using the Image Tool 3.0 software [30].

The quantification of mast cells/basophiles was made by counting 200 cells per slide, from predetermined contiguous microscopic fields.Mast cells were classified as “degranulated” and “not degranulated” according to the presence or not of histamine granules outside the cell, despite the degree of cytoplasm emptiness. To avoid interpretative bias, two independent observers did this procedure in blind. Results were treated together in statistical analysis.

### 2.8. Immunohistochemistry

The immunohistochemical procedure had the aim to put in evidence the adhesion molecules expressed by leukocytes (CD18) and endothelium (CD54), as well as to differentiate mononuclear phagocytes to other mononuclear cells into the infiltrate, including complementary evidence of their different stages of maturation, by using the CD163 marker.

Slices of 3 to 5 microns were cut and adhered on commercial silane-treated slides. Then, slides were deparaffinized in both, xylol and absolute alcohol bath, and washed in current tap water. All slides were heated in a citrate buffer bath (SIGMA, pH = 6.0), during 20 minutes, in ebullition, for antigen unmasking. After that, slides were washed in PBS, pH = 7.2 (SIGMA), dried with a soft paper, and cuts were delineated with Pap Pen (AbCam).

The endogenous peroxidase blocking was done by incubating slides in 5% H_2_O_2_ 1 : 4 in methanol PA (ISOFAR). Slides were washed in PBS (sigma) and incubated with normal horse serum (VECTOR) during 20 minutes, for blocking unspecific protein adsorption sites, then immediately incubated with the primary antibody (see Table 1) in a humid chamber, at 4°C, overnight.

In the next day, cuts were washed in PBS and incubated during 30 minutes with the secondary antibody conjugated to micropolymers coupled to peroxidase (VECTOR). Then, they were washed again and incubated with DAB (DAKO) for 3 seconds. After being washed in current tap water, all slides were stained with the Harris hematoxylin and mounted under a glass cover. Slides stained by hematoxylin-eosin method have done as reference to specific slides areas and to count polymorphonuclear cells (PMN), specifically neutrophils. In this case, the number of PMN cells per field was determined in 10 randomized fields, using 100x lens.

### 2.9. Quantification

**Figure 1:**
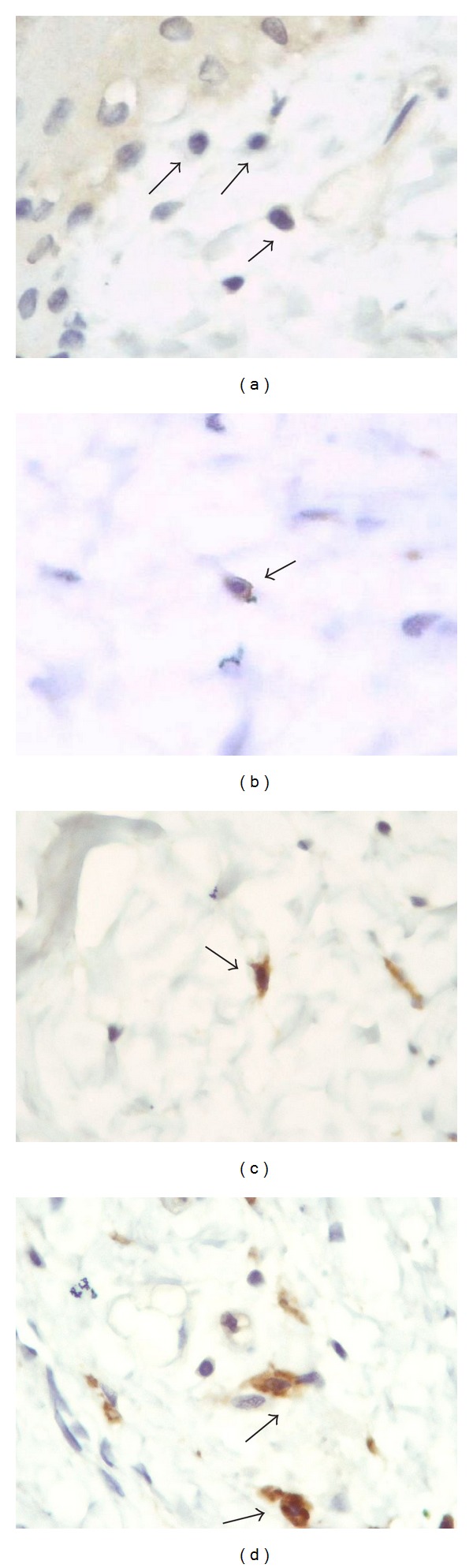
Photomicrographs of inflamed footpad of the offspring born to mothers treated with dexamethasone 15 cH. Animals were inoculated with 1% carrageenan into the footpad. Material stained by immunohistochemistry using polyclonal anti-CD163 (Serotec). (a) Negative mononuclear cells, (b) monocyte marked on the membrane, (c) monocyte marked on the cytoplasm, (d) macrophage marked on the cytoplasm. 100x objective.

 In the case of CD18, CD163, and MAC 387 markers, 10 randomized microscopic fields were observed per slide, using immersion objective (100x). The number of positive cells per field was recorded. For MAC 387, only mononuclear cells were considered. For CD18, counting procedures were done by two independent observers, in blind, considering both the total of positive cells and only the mononuclear positive cells. This proportion indicates the rate of mononuclear cell migration. For CD 163, different degrees of staining were considered, because the ED protein is expressed progressively, according to monocyte maturation into the inflammation site [31–33]. Then, quantification were organized in 4 criteria (Figure 1): (a) negative monocytes; (b) weak and irregular marking of cell membrane; (c) strong marking of membrane and cytoplasm; (d) large macrophage-shaped cells strongly positives to CD 163. For CD54, scores from 1 to 4 were attributed to each field, in order to differentiate the degree of intensity. To reduce the subjectivity, three independent observers participated in this counting procedure, in blind, according to [34].

### 2.10. Statistical Analysis

#### 2.10.1. *In Vivo* Step

The MANOVA test was used to compare the fourth initial groups, considering the basic toxicological parameters. The Student's *t*-test (for parametric variables) or the Mann-Whitney test (for nonparametric variables) was used for comparison of parameters from both UHD and CONT groups. The data regarding proportionality were analyzed by the Fisher test.

#### 2.10.2. Histological Step

The method first employed to evaluate results was the Bartlett test, in order to determinate the homogeneity of samples. Then, Student's *t*-test or Mann-Whitney's *t*-test was used, according to the results. To analyze negative-positive cell proportions in different groups, the *χ* ^2^ test was used.

Values of *P* ≤ 0.05 were considered as statistically significant. All statistical analysis was performed using the Instat 3.0 software.

### 2.11. Bioethics

The protocol was approved by the Research Bioethical Committee of UNIP (number 008/2009) and was according to the Brazilian Laws for Animal Protection.

## 3. Results

### 3.1. The P (Parent) Generation

**Figure 2:**
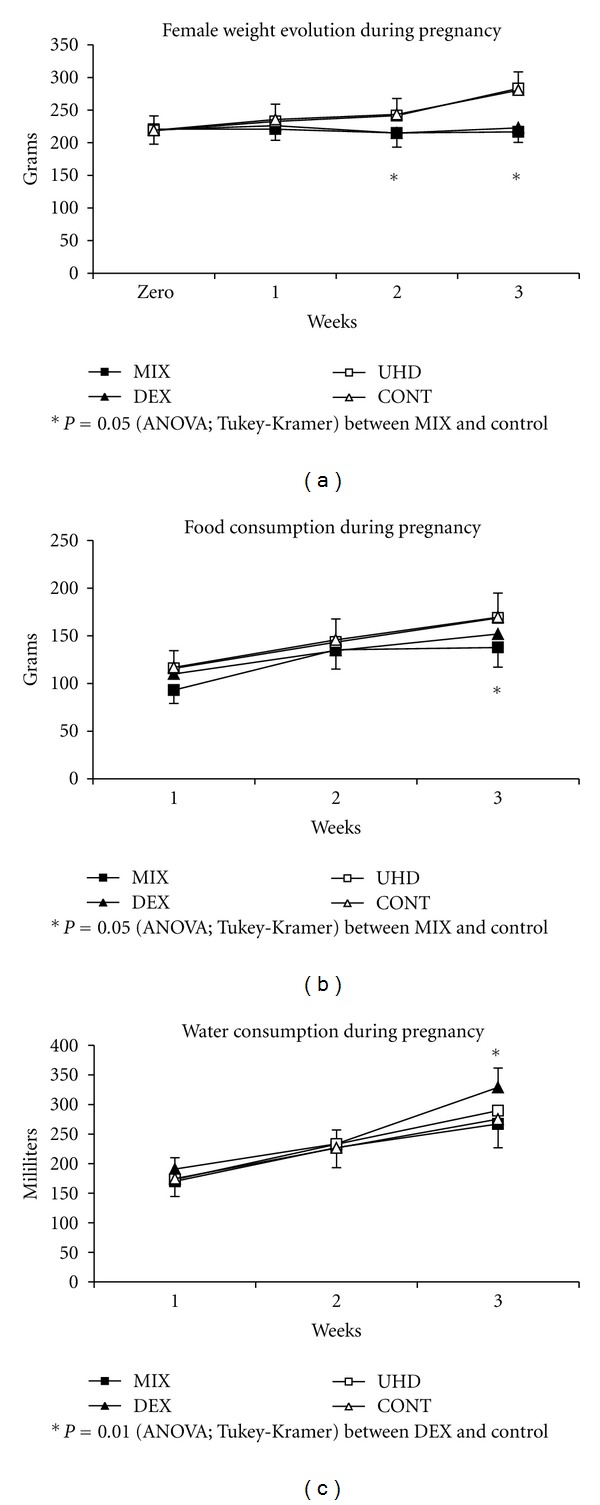
(a) Female weight evolution during pregnancy (grams); (b) food consumption during pregnancy (grams); (c) water consumption during gestation (mL). MIX = dexamethasone 15 cH + dexamethasone 4 mg/kg (UHD + DX); DEX = dexamethasone 4 mg/kg (DX); UHD = dexamethasone 15 cH; CONT = control (water). *MANOVA, *P* = 0.0002.

Data obtained from the first part of the study show smaller weigh gain in P generation females treated with dexamethasone 4 mg/kg, independently of the cotreatment with dexamethasone 15 cH (MIX and DEX groups). This result reflects the output of the gestation interruption in these groups (Figure 2(a)).

At the end of the treatment, both groups presented no significant mild reduction in food intake compared to UHD and CONT groups (Figure 2(b)). The DEX group (treated only with dexamethasone 4 mg/kg) presented little but not significant increase in water intake, a universal measure of toxic effects (Figure 2(c)).

At the end of gestation, no birth was observed in MIX and DEX groups, it means, despite the cotreatment with dexamethasone 15 cH, all females exposed to dexamethasone 4 mg/kg exhibited its classical toxic effect. At the necropsy, these females presented incidence of 100% of adrenal fascicular zone atrophy and 100% of embryo absorption. Pulmonary hemorrhage was seen in 17 to 42% of females from these groups (*P* = 0.03, Fisher test, comparing to UHD and CONT groups). Some females from UHD and CONT presented cannibalism without statistical significance (Table 2). These animals continued to be treated during lactation and no difference was seen concerning weigh evolution, food and water intake during this period.

The results show a discrete protective effect of dexamethasone 15 cH on toxic effects of pharmacological doses of the same drug in pregnant females, regarding of water consumption. The other parameters were according to the expected effect of corticoid intoxication during pregnancy. Thus, 100% of females treated with pharmacological doses of dexamethasone, despite the concomitant treatment with dexamethasone 15 cH, presented embryo absorption. In this case, only the F1 generation born to UHD and CONT mothers was analyzed for postnatal parameters.

### 3.2. The F1 (First Descendent) Generation

**Figure 3:**
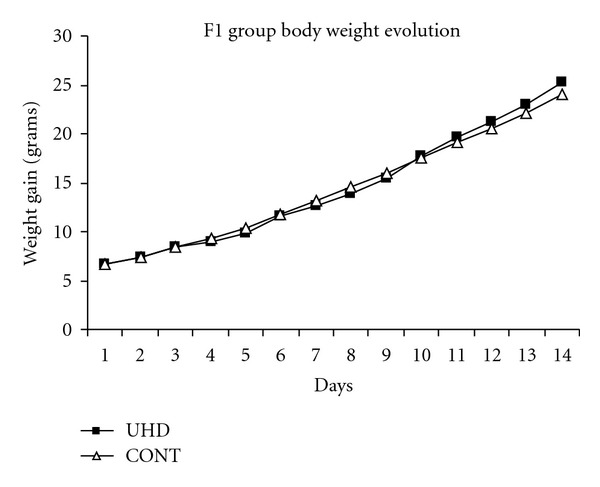
F1 generation weight gain evolution (grams). The 4 selected rats of each offspring were weighed together; so, there is no standard deviation and statistics for each time. UHD = dexamethasone 15 cH; CONT = control (water).

The homeopathic dexamethasone exposition did not alter parameters related to post-natal development of newborns (Table 3), except a mild not significant reduction of offspring weight gain in experimental group (UHD) in relation to control (CONT) between days 4 and 8 after birth, only (Figure 3).

**Figure 4:**
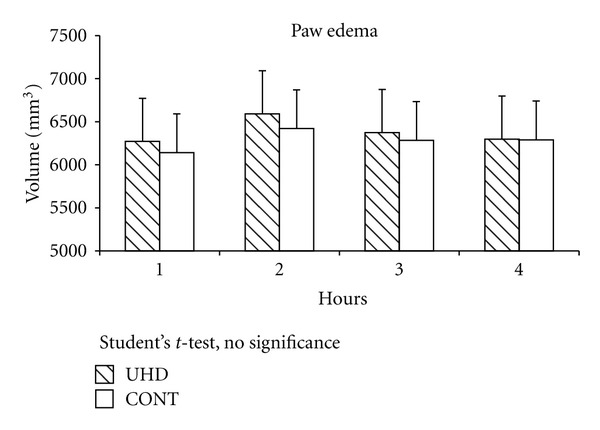
Paw edema after 1% kappa carrageenan (0.1 mL, sc). Millimeters. UHD = dexamethasone 15 cH; CONT = control (water). Student “*t*” test.

After 60 days, the F1 generation was inoculated with 1% carrageenan into the footpad, to evaluate its patterns of inflammation response face to an irritant stimulus. The edema curve was measured each hour, during 4 hours, and no difference between groups was seen, considering this macroscopic parameter (Figure 4).

**Figure 5:**
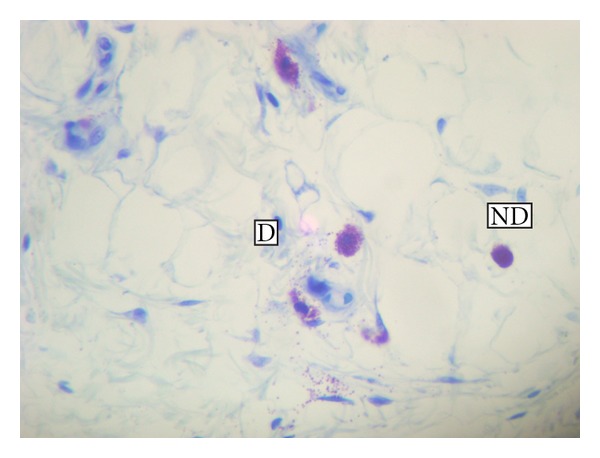
Photomicrographs of inflamed footpad of the offspring inoculated with 1% carrageenan (magnitude 400x). ND = not degranulated cell; D = degranulated cells.

After 4 hours of edema evaluation, rats were sacrificed and the histopathological analysis of inflamed subcutaneous tissue was done. The toluidine blue staining followed by histomorphometry revealed a significant difference in mast cell/basophile degranulation percentage between groups. Thus, rats born to mothers treated with dexamethasone 15 cH (UHD) presented more degranulated mast cells per field than control (Table 4, Figure 5).

**Figure 6:**
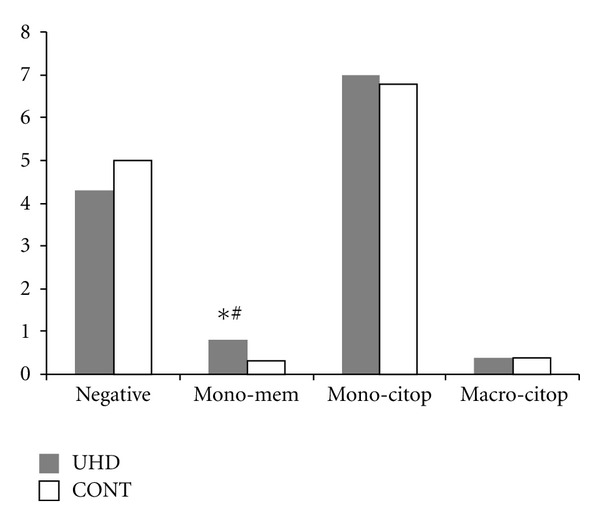
CD 163 positive monocytes and macrophages per slide (10 fields per slide), representing inflamed subcutaneous tissue of the carrageenan inoculated footpad from rats born to mothers treated with dexamethasone 15 cH (UHD) or water (CONT). Quantification was based on 4 categories of staining intensity: (a) negative monocytes; (b) irregular positivity of monocyte membranes; (c) intense cytoplasm positivity of monocytes; (d) intense cytoplasm positivity of macrophages. Columns represent mean. (#) *χ* ^2^ negative/positive ratio, *P* = 0.0001 in relation to control; (∗) Student's *t*-test, *P* = 0.0004 in relation to control.

After the HE staining method, a nonsignificant increase of neutrophils per field was seen in UHD group, associated to a significant increase in MAC 387 + mononuclear cells per field. A greater proportion between CD163 + monocytes and negative monocytes (Table 4, Figure 6) was also seen between groups, suggesting earlier expression of ED2 protein in experimental group during phagocyte activation.

No significance was seen in relation to specific CD18+ mononuclear cells per field, but a significant increase of total CD18+ leukocytes was seen in UHD group, in relation to control (CONT), indicating higher expression of beta-2 integrin in PMN cells than in mononuclear cells (Table 4). The intensity of CD54+ endothelial cells was not significant between groups (Table 4).

## 4. Discussion

The results show that no developmental variation is seen in rats born to mothers treated with dexamethasone 15 cH (10^−33^ M) during pregnancy and lactation but also suggest the existence of a subtle but measureable upregulation of acute inflammatory process in rats born to these mothers. This statement can be justified by histological and histomorphometric coherent parameters found in subcutaneous inflamed tissue, such as increase in mast cell degranulation, increase in PMN/mononuclear ratio, increase in CD18+ leukocytes, and early expression of ED2 protein (CD163) in migrated monocytes, indicating early phagocyte maturation. These parameters, mainly the mast cell degranulation and PMN cell migration, are generally inhibited by pharmacological concentrations of corticoids; curiously, the opposite effect was seen when animals were exposed to the 15 cH potency of dexamethasone. This fact refers to the opposite effect generally seen in the similia principle. In further studies, a deeper understanding about cytokines balance in these situations could be explored.

The whole data indicated changes of the pattern of inflammatory response in the high-diluted prenatal exposed offspring, in a sense of showing the “acute trend” in migrated leukocyte profile after an irritant stimulus, what is not seen in the control. This result reinforces the hypothesis of some kind of “vertical” (mother-fetus) transmission of information from high-diluted active substances, although no macroscopic parameter has been touched.

This study is pioneer in this field of knowledge and has some fundamental and applied implications. Under the fundamental research about high dilutions, these data point to some important aspects of the way of action of high-diluted products; it means changing the global configuration pattern of physiological parameters, instead of changing one or another specific marker involved in the process. Under the applied point of view, these results give useful information about putative consequences after the use of homeopathic preparations in breeding animals, especially in organic farms. According to the common sense, since there is no chemical residue in homeopathic remedies, the safety of these products would be absolute, regarding animal-derived food products. However, no trustful conclusion can be done “a priori,” whatever is the theme of study. In this case, an important window is opened, mainly related to the unknown long-term consequences of the use of homeopathic products in herds. This is not a simply toxic effect but a biological underexplored phenomenon that begins to be investigated. The scientific studies made under rigorous and systematic methodology are a sine-qua-non condition to the scientific development of the high-dilution field, as well in human, as well in veterinary medicine.

Although innovative and preliminary, the present study reflects original results and the plausibility to state the mother-fetus transmission of biological information from the theoretical above Avogadro's number high-diluted dexamethasone. The only previous result found in the literature about that is a paper published by our own group, using mice in a similar model [35]. However, in this case, a huge discrepancy of results is seen: although in mice, dexamethasone 15 cH interfered in the pregnancy evolution and increased cannibalism by mothers, in rats, only subtle effects were seen in F1 generation, concerning acute inflammation modulation. 

Abrupt changes in results according to the experimental context and variables are quite common in experimental studies about highly diluted endogenous substances. In [36], the effect of homeopathic thymulin was the opposite between experiments performed in summer or winter and this fact was observed in several repetitions, during two years. Other studies that present variations in output according to experimental context can be also found in the literature [37, 38]. In this case, the animal species was decisive for the output features, since both rats and mice experiments were tested for the same dose and potency of dexamethasone, in the same room conditions and both were performed during the Brazilian winter.

The effect of 0.4 mg/kg dexamethasone in young rat development is well known and is related to bone growth and calcification delay [27]. However, the treatment of pregnant females with lower doses (0.05 mg/kg) determines delicate changes in mother and fetus tissues, such as glycogen accumulation in placenta [28] and number of synapses, related to the expression of c-fos [29]. Repeated administration of glucocorticoid in low doses during gestation leads to the downregulation of its receptor in liver and hippocampus [39], which can be related to special metabolic parameters in these animals when adults, like hypertension and precocious puberty [40]. Recently, the epigenetic F1 and F2 two-generation transmission of glucocorticoid effects on cardiac disease risk was demonstrated after prenatal P generation exposition to this hormone. The authors point to DNA methylation as the possible mechanism of this multigenerational programming [41]. Beside the known effects of glucocorticoid in epigenetic phenomena, we still do not know how these mechanisms actually participate in the process involving very high dilutions of the same hormone.

The choice of using CD163 as a phagocyte maturation marker, beside MAC 387, has allowed the observation of detailed dynamics in migration and maturation of mononuclear cells into inflammatory site, since the maturation of monocytes in active macrophages is followed by the 15-fold increase in CD163 expression by these cells. The CD163 is a type I transmembrane glucoprotein (175 kD), also known as ED2. Its expression is related to the endocytosis of hemoglobin complexes and to the followed metabolism. Positive cells for ED2 protein have modulation role in inflammation and they can secret autocrine IL-10, which makes positive feedback with IL-4 and corticoids [31–33].

In this study, the dilutions of dexamethasone used were very high, above Avogadro's number—15 cH means 10^-33 ^M—and, even though, subtle modulation effects could be seen, regarding mast cell/basophile degranulation and inflammation cells infiltrate profile and maturation. This new perspective adds more information about the role of glucocorticoid in pregnancy and opens a wide range of research possibilities about homeopathy and its use in pregnant animals.

As conclusion, the proposed experimental model was able to demonstrate the mother-fetus transmission of homeopathic information, opening a new field of studies about homeopathy in pregnancy and its particular features in organic animal production.

## Figures and Tables

**Table 1:** Immunohistochemistry used markers (adapted from KAWAKAMI, 2011) [29].

Marker	Cell	Source	Molecular target	Clone	Origin/target (species)	Dilution
Anti-CD54	Activated endothelial cell	Serotec	ICAM 1	1A29	Mouse-rat	1 : 10 (5 *μ*g/mL)
Anti-CD18	Leukocytes	Serotec	Beta-2 integrin	WT.3	Mouse-rat	1 : 10 (5 *μ*g/mL)
Anti-CD163	Activated macrophages	Serotec	Surface glycoprotein (ED2)	ED2	Mouse-rat	1 : 10 (5 *μ*g/mL)
Anti-MAC 387	Monocytes, macrophages, and polymorphonuclear	AbCAM	Intracytoplasmatic protein (calprotectin)	polyclonal	Rabbit-rat	1 : 20 (50 *μ*g/mL)

**Table 2:** Percentage of macroscopic lesions in MIX = dexamethasone 15 cH + dexamethasone 4 mg/kg; DEX = dexamethasone 4 mg/kg; UHD = dexamethasone 15 cH; CONT = control (water). ^∗^ *P* = 0.03; ^∗∗^ *P* = 0.0001, in relation to MIX. Fisher's test.

Macroscopic lesions	MIX Dexa 4 mg/kg + Dexa 15 cH	DEX Dexa 4 mg/kg	UHD Dexa 15 cH	CONT vehicle
Embryo absorption	100^∗∗^	100^∗∗^	10	20
Cannibalism evidence (percentage of dead newborns found in the cage)	—	—	13	6
Cannibalism evidence (percentage of mothers that showed this behavior)	—	—	41	25
Adrenal fascicular zone atrophy	83	100	08	0
Diffuse lung hemorrhage	42^∗^	17	0	0

**Table 3:** Parameters of individual postnatal development (days). MIX = dexamethasone 15 cH + dexamethasone 4 mg/kg; DEX = dexamethasone 4 mg/kg; UHD = dexamethasone 15 cH; CONT = control (water). Student's *t*-test. Mean/SD.

Parameters (days)	UHD	CONT
Pinna (ear) opening	2.43^#^/0.55^##^	2.38/0.49
Eyes opening	12.32/1.39	12.16/0.98
Vagina opening	40.05/0.23	40.00/0.00
Superior incisive teeth dehiscence	9.79/1.56	9.98/1.53
Superior incisive teeth dehiscence	10.63/1.21	10.93/1.53
Fur covering	1.01/0.11	1.00/0.00
Testicle dehiscence	4.00/0.00	4.00/0.00
Postural reflex learning	12.31/2.15	12.85/2.30

^#^mean; ^##^standard deviation.

**Table 4:** Number of inflammatory cells in the subcutaneous tissue of the footpaw inoculated with 1% kappa carrageenan. PMN cells were counted using HE stained slides. Immuno-histochemistry was used to identify different stages of phagocyte maturation (CD 163) and the number of mononuclear phagocytes per field (MAC 387). Adhesion molecules CD18 (beta integrin) and CD54 (ICAM 1) were marked in leukocytes and endothelial cells. The intensity of CD54 marked cells was measured by pre-defined scores measured by three independent observers. The proportion of degranulated mast cells (N/200 total counted cells) is represented by percentage. The values are represented by mean (*χ*) and standard deviation (SD). ^∗^Student “*t*” test, *P* ≤ 0.01 in relation to control (CONT); ^#^ *χ* ^2^, *P* = 0.001 in relation to negative/positive ratio of control group (CONT).

	Dexamethasone 15 cH	Control
Eosin/hematoxylin (PMN)	*χ* = 0.54 SD = 2.114	*χ* = 0.33 SD = 1.124
MAC 387 + (Macrophages)	*χ* = 0.58^∗^ SD = 0.923	*χ* = 1.40 SD = 1.725
CD163 + (negative monocytes)	*χ* = 4.28^#^ SD = 5.386	*χ* = 4.99 SD = 5.741
CD163 + Membrane-marked monocytes	*χ* = 0.790^∗^ SD = 1.664	*χ* = 0.321 SD = 0.919
CD163 + cytoplasm marked monocytes	*χ* = 7.00 SD = 5.399	*χ* = 6.76 SD = 6.550
CD163 + cytoplasm marked macrophages	*χ* = 0.533333 SD = 0.787863	*χ* = 0.491525 SD = 0.90342
CD 18 + (positive mononuclear cells)	*χ* = 1.55 SD = 1.559429	*χ* = 1.922222 SD = 3.638829
CD 18 + (negative mononuclear cells)	*χ* = 8.944444 SD = 3.724686	*χ* = 10.21111 SD = 4.384586
CD 18 + (positive total leukocytes)	*χ* = 18.74^∗^ SD = 8.15	*χ* = 15.41 SD = 7.68
CD 18 + (negative total leukocytes)	*χ* = 0.75 SD = 1.06	*χ* = 0.55 SD = 0.99
CD 54 + (scores)	*χ* = 2.33 SD = 1.057	*χ* = 2.41 SD = 1.057
Degranulated mast cells (*n*/200 counted cells)	9.5/200 (4.75%)^#^	5.9/200 (2.95%)
